# Supplementary material for: Elemental Sulfur Inhibits Yeast Growth via Producing Toxic Sulfide and Causing Disulfide Stress
Source: Antioxidants (Basel). 2022 Mar 17;11(3):576. doi: 10.3390/antiox11030576 (PMC8945482; doi:10.3390/antiox11030576)
Supplement: Supplementary file 1 [file antioxidants-11-00576-s001.zip › antioxidants-1616798-supplementary.pdf]

Supplementary Materials for

Elemental Sulfur Inhibits Yeast Growth via Producing Toxic Sulfide and Inducing Disulfide Stress

Tianqi Wang[1], Yuqing Yang[1], Menghui Liu[1], Honglei Liu[1], Huaiwei Liu[1], Yongzhen Xia[1*], Luying Xun[1,2*]

[1]State Key Laboratory of Microbial Technology, Shandong University, Qingdao, 266237, China

[2]School of Molecular Biosciences, Washington State University, Pullman, WA, 99164-7520, USA

* To whom correspondence should be addressed to L. Xun. Tel: +1-509-335-2787; Email: luying_xun@vetmed.edu.edu; Correspondence may also be addressed to Y. Xia. Tel: +86-532-58631572; Email: xiayongzhen2002@sdu.edu.cn.

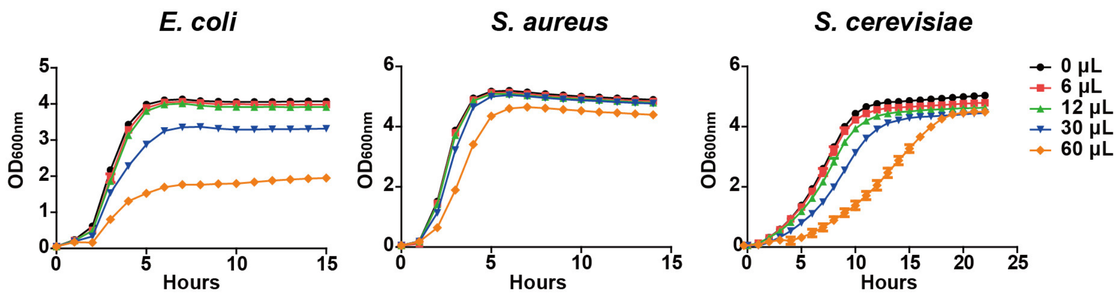

Figure S1. The effect of acetone on the growth of *E. coli*, *S. aureus*, and *S. cerevisiae*. Acetone was added to cultures at 0, 6, 12, 30, and 60 μL per mL, corresponding to adding 0, 100, 200, 500, and 1000 μM elemental sulfur dissolved in acetone, as reported in Fig. 1. The cultures were kept on a shaker under optimal growth conditions. The initial OD_{600nm} was 0.05. Data are averages of three parallel experiments with standard deviations.

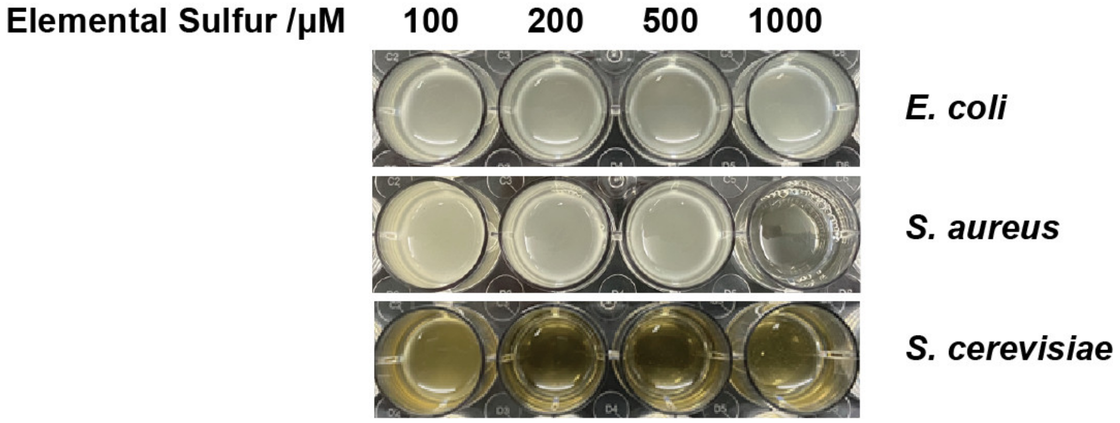

Figure S2. The minimal inhibitory concentration (MIC) assay of *E. coli*, *S. aureus,* and *S. cerevisiae*. The strains were inoculated to the proper media with different elemental sulfur concentrations with an OD_{600nm} of 0.05 and incubated with shaking. Results shown were after 12 h inoculation.

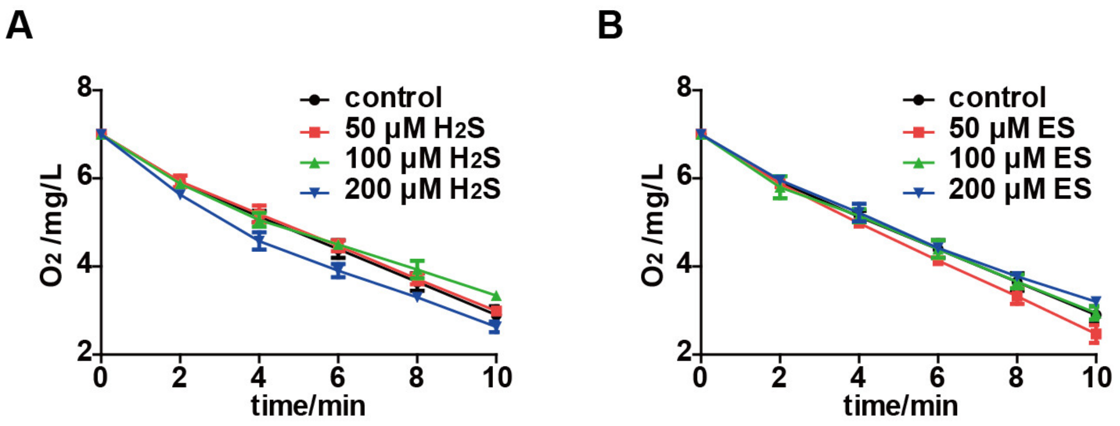

Figure S3. The effects of H_2S and elemental sulfur on O_2 consumption by *E. coli*.

(A) Oxygen consumption by *E. coli* after incubating with different concentrations of H_2S for 10 min. (B) Oxygen consumption after incubating with different concentrations of elemental sulfur for 10 min. The resting cells with an OD_{600nm} at 2.0 were incubated in 100 mM PBS buffer. 2% Glucose was added to initiate the test. Data are averages of three parallel experiments with standard deviations. ES, elemental sulfur.
